# Foot and knee deformities in relation to functional limitations and incident osteoarthritis: A prospective cohort study

**DOI:** 10.1016/j.afos.2024.08.002

**Published:** 2024-09-11

**Authors:** Jonathan K.L. Mak, Kathryn Choon Beng Tan, Janus Siu Him Wong, Martin Man Ho Chung, Ching-Lung Cheung

**Affiliations:** a Department of Pharmacology and Pharmacy, The University of Hong Kong, Hong Kong SAR, China; b Department of Medical Epidemiology and Biostatistics, Karolinska Institutet, Stockholm, Sweden; c Department of Medicine, School of Clinical Medicine, The University of Hong Kong, Hong Kong SAR, China; d Department of Orthopaedics and Traumatology, School of Clinical Medicine, The University of Hong Kong, Hong Kong SAR, China; e Laboratory of Data Discovery for Health (D24H), Hong Kong Science Park, Pak Shek Kok, Hong Kong SAR, China

**Keywords:** Foot pain, Foot disorders, Knee alignment, Falls, Function, Osteoarthritis

## Abstract

**Objectives:**

This study aimed to investigate the relationships of foot and leg symptoms, structure, and function with functional limitations and osteoarthritis (OA).

**Methods:**

We included 1253 participants (mean age 58.1 years) from the Hong Kong Osteoporosis Study who completed an examination on foot posture, function, pain, and presence of deformities such as hallux valgus and varus knee. Using logistic regression, we estimated cross-sectional associations of each foot and knee problem with functional outcomes (slow walking speed, self-reported falls, and functional limitations) and OA. Through linkage to electronic health records, we further examined their associations with incident OA over 8 years using Cox models. All models were adjusted for age, sex, and body mass index.

**Results:**

The prevalence of hallux valgus, foot pain, and varus knee were 33.1%, 35.1%, and 25.8%, respectively. Planus foot posture was associated with varus knee, and pronated foot function was associated with hallux valgus. Of the assessed foot problems, only foot pain showed significant associations with functional outcomes, including functional limitations and recurrent falls. Foot pain was also associated with prevalent OA at baseline but not incident OA. Meanwhile, we observed a 3-times increased risk of incident OA associated with varus knee (95% CI = 1.48–6.10), and this association was particularly seen in older adults, women, and obese individuals.

**Conclusions:**

In community-dwelling Chinese adults, foot pain, but not the reported foot deformities, is associated with functional limitations and falls, while varus knee is associated with incident OA.

## 1 Introduction

Foot and leg symptoms, structure, and function, such as foot pain, flat foot, hallux valgus/bunions, and knee malalignment, are common in the general population, with estimated prevalence of 13%–36% for foot pain [1] and 19% for hallux valgus [2]. These problems are usually associated with restricted joint movement and impaired balance, thereby increasing the risks of falls, as well as functional and mobility limitations, particularly in older adults [[3], [4], [5], [6]]. However, their relationship with osteoarthritis (OA) of the knee is less clear.

OA typically affects the knee joint and is associated with increased risks of disability and mortality [7,8]. Risk factors of OA include advancing age, female sex, obesity, genetics, and joint injury [9]. In addition, biomechanical factors such as malalignment of the knee joint in either varus (bowed legs) or valgus (knock knee) directions can lead to the progression of knee OA [10,11]. Nevertheless, since most previous studies were cross-sectional in nature, there is less evidence on whether knee deformities could also be associated with incident OA [10,11]. While some studies suggested that varus alignment may increase the risk of knee OA development, especially in overweight and obese individuals [[12], [13], [14], [15]], others reported a null association [8]. In addition, although OA patients are more likely to have a pronated foot function [16] and hallux valgus [17], there is a paucity of longitudinal data to test whether foot deformities or foot pain may also be risk factors of incident OA [18]. Particularly, prior studies were mostly confined to Caucasian populations [4], with relatively limited evidence on the prevalence and associated outcomes of foot and knee problems in other populations, such as in Asia where foot disorders are common [2].

To address these knowledge gaps, we performed a cohort study to describe the prevalence of foot and knee deformities in a sample of community-dwelling Chinese adults and investigate their relationships with functional limitations and incident OA.

## 2 Methods

### 2.1 Study population

Participants were from the follow-up study (2015–2019) of the Hong Kong Osteoporosis Study (HKOS). Details of the HKOS have been described previously [19]. Briefly, between 1995 and 2010, over 9000 community-dwelling Southern Chinese women and men completed structured questionnaires and provided clinical and biomedical data at the baseline assessment. A full-scale follow-up study was later conducted between June 2015 and August 2019, in which 1386 participants provided musculoskeletal disease-related data, including gait and foot measures, demographic, clinical and biomedical data. The HKOS participants are also linked to the Clinical Data Analysis and Reporting System, the territory-wide electronic health record database that captures both inpatient and outpatient records and covers > 80% of hospital admissions in Hong Kong. This study was approved by the Institutional Review Board of The University of Hong Kong/HA HKW, Hong Kong Special Administrative Region, China. Informed consent has been obtained from all participants prior to data collection.

For this analysis, we excluded those with missing data on the covariates (N = 1), foot measures (N = 130), and functional limitations (N = 2), leaving N = 1253 in the analytic sample.

### 2.2 Foot and knee measures

Foot posture and dynamic foot function were examined by trained examiners using a Tekscan MatScan foot pressure mat during quiet standing and walking at a self-selected pace, respectively [20]. Foot posture was assessed by a modified arch index (MAI), which was derived as the pressure in the middle-third of a foot divided by the total pressure under the foot during quiet standing [21]. Dynamic foot function was assessed by the center of pressure excursion index (CPEI), which was derived as the percentage excursion of the center of pressure during walking [22]. As each individual has two values of MAI and CPEI (one value in each foot), we followed previous work and took the more extreme values relative to the median in the analysis [20]. Foot posture and foot function were categorized into three groups based on the sex-specific quintiles of MAI and CPEI [20]. The bottom and top 20% of the MAI values were considered as cavus/high arch (women: < 0.105; men: < 0.107) and planus/low arch (women: > 0.235; men: > 0.247), respectively, and the middle 60% was considered as the normal foot posture [20]. Similarly, dynamic foot function was categorized into three groups based on the CPEI values, where the bottom 20% was defined as over-pronated and everted hindfoot (women: < 11; men: < 15), the top 20% as over-supinated and inverted hindfoot (women: > 28; men: > 30), and the middle 60% as the normal foot function [20].

Other foot and knee deformities, including hallux valgus and varus knee deformity, were evaluated based on clinical observations by trained examiners. Varus knee was defined as intercondylar distance > 3 cm [23,24]. Hallux valgus was evaluated using a validated instrument with five drawings of each foot representing five degrees of severity of hallux valgus [25]. The two least severe grades were defined as absent, and the other three more severe grades were defined as present. Consistent with previous studies [3,6], we defined generalized foot pain in one or both feet using the self-reported question: “On most days do you have pain, aching, or stiffness in either of your feet?”

### 2.3 Outcome measures

Four functional outcomes were assessed, including walking speed, mobility limitation, limitations in activities of daily living (ADL), and recurrent falls. Walking speed (meter/second) was measured using a 6-m walking test, and we defined slow walking speed as < 0.8 m/s [26]. Mobility limitation was self-reported and defined as having at least some difficulties in walking 1 km or climbing 10 steps [27]. ADL limitation was defined as having difficulties in any of the self-reported ADL items, including dressing, bathing, eating, transferring, toileting, and taking medications. Recurrent falls were self-reported and defined as falling ≥ 2 times in the past year.

OA was ascertained based on both self-reported medical history of OA and electronic health records in the CDARS using the International Classification of Diseases, 9th Revision (ICD-9) code 715. We also assessed knee OA using ICD-9 codes 715.16, 715.26, 715.36, and 715.96 [28]. In the longitudinal analysis, we included only those without a self-reported or diagnosed OA at baseline (N = 1126). Participants were then followed until the first date of OA diagnosis, death, or end of follow-up (January 15, 2024), whichever came first.

### 2.4 Statistical analysis

Participant characteristics were summarized and compared by sex using *t*-tests for continuous variables and *χ*^2^ tests for categorical variables. We examined the cross-sectional associations of foot posture and function with hallux valgus, foot pain, varus knee using multivariable logistic regression models adjusted for age, sex, and body mass index (BMI).

For each foot and leg symptoms, structure, and function (abnormal foot posture, abnormal foot function, hallux valgus, foot pain, and varus knee), we assessed their cross-sectional associations with the outcome measures using logistic regression models. Cox models were used in the longitudinal analysis to assess the associations with incident OA. Moreover, we assessed whether the foot and knee measures were associated with knee OA in particular. The proportional-hazards assumption was verified using Schoenfeld residuals. All the regression models were adjusted for age, sex, and BMI.

For the foot and/or leg symptoms, structure, and function that were significantly associated with incident OA, we further performed subgroup analyses to test if the associations differ by age (< 65 vs. ≥ 65 years), sex (women vs. men), and BMI (normal < 23 kg/m^2^, overweight 23–24.9 kg/m^2^, obese ≥ 25 kg/m^2^, as per the Asia-Pacific cutoff points) [29]. To minimize reverse causality due to an undiagnosed OA at baseline, we also performed a sensitivity analysis by excluding the first 3 years of follow-up in the longitudinal analysis.

All analyses were performed in R version 4.3.2. A two-sided P < .05 was considered as statistically significant.

## 3 Results

Of the 1253 included HKOS participants, the mean age was 58.1 years (standard deviation 11.7, range 27–87) and 79.4% were women. The prevalence of hallux valgus, foot pain, and varus knee were 33.1%, 35.1%, and 25.8%, respectively (Table 1). Adjusting for age, sex, and BMI, pronated (odds ratio [OR] = 1.40, 95% confidence interval [CI] = 1.01–1.93) and supinated foot functions (OR = 0.57, 0.40–0.81) were associated with increased and decreased odds of hallux valgus, respectively, while planus foot posture (flat foot) was associated with increased odds of varus knee deformity (OR = 1.50, 95% CI = 1.06–2.10) (Table 2). Hallux valgus also tended to be more common among those with a more planus foot posture, as indicated by the increased odds associated with a higher MAI (OR per SD increase = 1.22, 95% CI = 1.06–1.39). On the other hand, a higher CPEI (indicating more supinated foot function) was associated with reduced odds of foot pain (Table 2).

**Table 1:** Characteristics of study participants by sex.

Variable	Total (N = 1253)	Women (N = 995)	Men (N = 258)	P^a^
Age, years, mean ± SD	58.1 ± 11.7	57.9 ± 11.5	59.1 ± 12.7	0.14
BMI, kg/m^2^, mean ± SD	23.3 ± 3.7	23.2 ± 3.8	23.8 ± 3.3	0.029
MAI, mean ± SD	0.17 ± 0.08	0.17 ± 0.08	0.18 ± 0.08	0.05
Foot posture, N (%)				0.97
Normal	766 (61.1)	608 (61.1)	158 (61.2)	
Cavus (high arch)	248 (19.8)	196 (19.7)	52 (20.2)	
Planus (low arch)	239 (19.1)	191 (19.2)	48 (18.6)	
CPEI, mean ± SD	20.5 ± 9.0	19.7 ± 8.8	23.7 ± 8.8	< 0.001
Foot function, N (%)				0.20
Normal	821 (65.5)	664 (66.7)	157 (60.9)	
Pronated	218 (17.4)	168 (16.9)	50 (19.4)	
Supinated	214 (17.1)	163 (16.4)	51 (19.8)	
Hallux valgus, N (%)	415 (33.1)	364 (36.6)	51 (19.8)	< 0.001
Foot pain, N (%)	440 (35.1)	380 (38.2)	60 (23.3)	< 0.001
Varus knee, N (%)	323 (25.8)	234 (23.5)	89 (34.5)	< 0.001
Slow walking speed, N (%)	47 (3.8)	41 (4.1)	6 (2.3)	0.24
Mobility limitation, N (%)	250 (20.0)	215 (21.6)	35 (13.6)	0.005
ADL limitation, N (%)	38 (3.0)	31 (3.1)	7 (2.7)	0.90
Recurrent falls in past year, N (%)	65 (5.2)	55 (5.5)	10 (3.9)	0.36
Prevalent OA, N (%)	127 (10.1)	111 (11.2)	16 (6.2)	0.025
Incident OA during follow-up^b^, N (%)	33 (2.9)	28 (3.2)	5 (2.1)	0.49
Died during follow-up^b^, N (%)	20 (1.8)	11 (1.2)	9 (3.7)	0.021

BMI, body mass index; CPEI, center of pressure excursion index; MAI, modified arch index; OA, osteoarthritis; SD, standard deviation.

a P-values were based on *t*-tests for continuous variables and *χ*^2^ tests for categorical variables.

b Only individuals without a prevalent osteoarthritis at baseline were included for the longitudinal analysis (N = 1126). 25 of the 33 incident OA cases were classified as knee OA.

**Table 2:** Cross-sectional associations of foot posture and function with other foot and knee problems, adjusting for age, sex, and BMI.

Measure	Hallux valgus		Foot pain		Varus knee	
	OR (95% CI)	P	OR (95% CI)	P	OR (95% CI)	P
Foot posture						
MAI (per SD increase)	1.22 (1.06, 1.39)	0.004	0.90 (0.79, 1.03)	0.13	1.26 (1.10, 1.46)	0.001
Cavus (vs. normal)	0.89 (0.64, 1.24)	0.49	1.08 (0.79, 1.48)	0.63	0.89 (0.62, 1.25)	0.50
Planus (vs. normal)	1.34 (0.97, 1.85)	0.07	0.84 (0.61, 1.16)	0.30	1.50 (1.06, 2.10)	0.021
Foot function						
CPEI (per SD increase)	0.75 (0.66, 0.85)	< 0.001	0.87 (0.77, 0.98)	0.032	1.01 (0.88, 1.15)	0.91
Pronated (vs. normal)	1.40 (1.01, 1.93)	0.043	0.91 (0.66, 1.26)	0.58	1.14 (0.80, 1.60)	0.46
Supinated (vs. normal)	0.57 (0.40, 0.81)	0.002	0.77 (0.58, 1.12)	0.12	1.08 (0.76, 1.52)	0.65

CPEI, center of pressure excursion index; CI, confidence interval; MAI, modified arch index; OR, odds ratio; SD, standard deviation.

Table 3 shows the association between each foot and knee problem with functional limitations. After adjusting for age, sex, and BMI, foot pain was significantly associated with higher odds of mobility limitation (OR = 2.07, 1.54–2.77), ADL limitation (OR = 1.97, 1.01–3.86), and recurrent falls (OR = 1.88, 1.13–3.15). Participants with varus knees also had a significantly slower walking speed (OR = 2.75, 95% CI = 1.88, 3.48). Foot posture, function, and hallux valgus were not significantly associated with these functional outcomes.

**Table 3:** Cross-sectional association of foot and knee problems with functional limitations, adjusting for age, sex, and BMI.

Measure	Slow walking speed		Mobility limitation		ADL limitation		Recurrent falls	
	OR (95% CI)	P	OR (95% CI)	P	OR (95% CI)	P	OR (95% CI)	P
Foot posture								
MAI (per SD increase)	1.21 (0.90, 1.62)	0.19	0.96 (0.82, 1.12)	0.63	0.87 (0.60, 1.24)	0.47	0.95 (0.71, 1.24)	0.71
Cavus (vs. normal)	0.41 (0.10, 1.20)	0.59	0.80 (0.52, 1.20)	0.29	1.60 (0.64, 3.66)	0.30	0.50 (0.20, 1.05)	0.09
Planus (vs. normal)	0.82 (0.39, 1.65)	0.15	0.82 (0.56, 1.19)	0.31	1.54 (0.67, 3.40)	0.29	0.75 (0.36, 1.45)	0.42
Foot function								
CPEI (per SD increase)	1.02 (0.76, 1.36)	0.87	1.04 (0.90, 1.20)	0.58	1.25 (0.91, 1.69)	0.16	1.16 (0.90, 1.48)	0.24
Pronated (vs. normal)	1.64 (0.78, 3.32)	0.10	1.23 (0.82, 1.80)	0.31	0.75 (0.22, 2.00)	0.60	0.55 (0.21, 1.22)	0.18
Supinated (vs. normal)	1.92 (0.85, 4.05)	0.17	1.24 (0.85, 1.79)	0.26	1.86 (0.86, 3.81)	0.10	1.51 (0.82, 2.69)	0.17
Hallux valgus	1.51 (0.83, 2.78)	0.18	1.13 (0.83, 1.52)	0.44	0.88 (0.43, 1.74)	0.72	1.35 (0.79, 2.26)	0.26
Foot pain	1.65 (0.90, 3.03)	0.10	2.07 (1.54, 2.77)	<0.001	1.97 (1.01, 3.86)	0.046	1.88 (1.13, 3.15)	0.015
Varus knee	2.75 (1.88, 3.48)	0.046	1.23 (0.89, 1.70)	0.21	0.61 (0.25, 1.29)	0.22	1.42 (0.81, 2.41)	0.21

CPEI, center of pressure excursion index; CI, confidence interval; MAI, modified arch index; OR, odds ratio; SD, standard deviation.

As shown in Table 4, varus knee (OR = 1.66, 95% CI = 1.09–2.51) and foot pain (OR = 1.47, 1.00–2.17) were associated with prevalent cases of OA at baseline. We further examined longitudinal associations between foot problems and incident OA over a median follow-up time of 7.0 years (range 0.6–8.6). A total of 20 individuals died during follow-up, and 33 incident OA cases were documented, of which 25 (75.8%) were knee OA. Of the assessed foot and knee problems, only varus knee was significantly associated with an elevated risk of any OA (hazard ratio [HR] = 3.00, 95% CI = 1.48–6.10), as well as knee OA (HR = 3.11, 95 % CI = 1.37–7.04) (Table 4). In the subgroup analysis, the association between varus knee and incident OA was significant only in older adults aged ≥ 65 years, women, and individuals with a BMI ≥ 25 (Table 5). Furthermore, this association remained significant in the sensitivity analysis after excluding diagnoses occurring in the first 3 years of follow-up (HR = 3.70, 95% CI = 1.52–9.00).

**Table 4:** Cross-sectional and longitudinal associations of foot and knee problems with osteoarthritis, adjusting for age, sex, and BMI.

Measure	Prevalent OA		Incident OA^a^		Incident knee OA^a^	
	OR (95% CI)	P	HR (95% CI)	P	HR (95% CI)	P
Foot posture						
MAI (per SD increase)	0.94 (0.77, 1.15)	0.56	1.14 (0.82, 1.60)	0.44	1.14 (0.77, 1.67)	0.52
Cavus (vs. normal)	0.93 (0.50, 1.62)	0.80	1.07 (0.40, 2.88)	0.90	1.23 (0.40, 3.78)	0.72
Planus (vs. normal)	0.89 (0.55, 1.42)	0.64	0.77 (0.33, 1.86)	0.57	0.93 (0.35, 2.46)	0.87
Foot function						
CPEI (per SD increase)	1.15 (0.95, 1.39)	0.14	1.05 (0.75, 1.46)	0.78	1.10 (0.75, 1.60)	0.63
Pronated (vs. normal)	0.55 (0.28, 1.02)	0.07	0.70 (0.24, 2.06)	0.52	1.01 (0.33, 3.08)	0.99
Supinated (vs. normal)	1.14 (0.70, 1.80)	0.59	1.06 (0.45, 2.48)	0.90	1.35 (0.52, 3.49)	0.54
Hallux valgus	1.18 (0.79, 1.75)	0.42	1.18 (0.58, 2.38)	0.64	0.76 (0.32, 1.78)	0.52
Foot pain	1.47 (1.00, 2.17)	0.049	1.57 (0.79, 3.13)	0.20	1.11 (0.50, 2.48)	0.80
Varus knee	1.66 (1.09, 2.51)	0.017	3.00 (1.48, 6.10)	0.002	3.11 (1.37, 7.04)	0.007

CPEI, center of pressure excursion index; CI, confidence interval; HR, hazard ratio; MAI, modified arch index; OA, osteoarthritis; OR, odds ratio; SD, standard deviation.

a Only individuals without a prevalent osteoarthritis at baseline were included for the analysis of incident OA (N = 1115).

**Table 5:** Subgroup analysis on the association between varus knee with incident osteoarthritis, adjusting for age, sex, and BMI.

Subgroup	Number of cases	HR (95% CI)	P
Age			
< 65 years (N = 812)	7 (1.2%)	1.78 (0.44, 6.78)	0.42
≥ 65 years (N = 441)	26 (4.9%)	4.08 (1.68, 9.94)	0.002
Sex			
Women (N = 884)	28 (3.2%)	3.17 (1.48, 6.82)	0.003
Men (N = 242)	5 (2.1%)	1.67 (0.27, 10.35)	0.58
BMI			
Normal, < 23 kg/m^2^ (N = 579)	10 (1.7%)	1.66 (0.47, 5.88)	0.44
Overweight, < 23–24.9 kg/m^2^ (N = 247)	6 (2.4%)	2.78 (0.53, 14.53)	0.23
Obese, ≥ 25 kg/m^2^ (N = 300)	17 (5.7%)	5.25 (1.97, 14.02)	< 0.001

BMI, body mass index; CI, confidence interval; HR, hazard ratio.

## 4 Discussion

There has been limited population-based evidence on the associations between foot and leg symptoms, structure, and function with OA, especially in non-European populations. In this study, we performed an examination of various foot and leg problems in a large cohort of community-dwelling Chinese adults. Our results showed that [1] hallux valgus and varus knee were more common among those with over-pronated foot function and planus foot posture, respectively [2]; foot pain, but not other common foot disorders, was associated with functional limitations and prevalent OA; and [3] varus knee deformity was associated with a significantly increased risk of incident OA, particularly in older adults, women, and obese individuals.

It has been reported that the prevalence of hallux valgus is higher in Asia (22.0%) than in Europe (18.4%) or North America (16.1%) [2]. In our sample of younger and older Chinese adults, foot problems are also prevalent, with 33.1% having hallux valgus and 35.1% having foot pain. Similar to a previous study [30], we also found that a more pronated foot function was associated with increased odds of hallux valgus. However, although prior research has suggested a link between planus foot posture and pronated foot function with foot pain [31], this was not observed in our study. This could be partly due to differences in the population, where both the mean MAI and CPEI values were higher in our sample (0.17 and 20.5) compared to the Framingham Foot Study (0.12 and 13.9) [20]. To our knowledge, this is also the first large-scale study reporting MAI and CPEI in an Asian population.

Previous studies, mostly conducted in the US and Australia, have shown that foot pain is associated with recurrent falls [3,4,32,33] and mobility limitation [6,34]. Adding to the literature, we showed that foot pain was not only associated with recurrent falls, but also with other functional limitations such as impairments in mobility and ADL in Chinese adults. Additionally, similar to our findings, a weaker or null association has been found in the literature between foot deformities, such as hallux valgus and planus (flat foot) or cavus (high arch) foot posture, and worse functional status or slower walk times [4,34], indicating that many of these foot disorders may not require special medical attention if they are not painful.

Only a few longitudinal studies have examined whether knee malalignment is a risk factor for incident OA other than OA progression [10,11], and conflicting results have been reported [8,[12], [13], [14], [15]]. In this study, we found a strong association between varus knee deformity and incident OA, especially in older adults aged ≥ 65 years, overweight or obese individuals, and women. These results are in line with a previous study that observed a significant association between varus knees and incident knee OA only in overweight and obese persons [12]. Similarly, another study found that varus knee was associated with the development of radiographic knee OA among overweight and obese women [15]. The increased risk of incident knee OA associated with varus knees could be explained by an increased loading [35] and cartilage damage of the medial compartment [36], especially in obese individuals, leading to an accelerated degeneration of the knee joint. It is however important to note that we did not have the actual measurement of mechanical alignment, therefore, our results should be interpreted with caution. On the other hand, while some studies suggested that foot/ankle pain may be associated with incident knee OA [18,37], we only observed a significant association between foot pain and prevalent OA at baseline, but not incident OA, which could be due to differences in our OA definition and population characteristics, or that OA is indeed more related to ankle rather than foot pain [18].

The strengths of this study include a well-characterized cohort of younger and older adults who have validated foot posture, function, and pain measures available. The linkage to the territory-wide electronic health records also allowed us to examine the longitudinal associations between various foot and leg problems and the risk of incident OA, thereby reducing potential reverse causation. Nevertheless, several limitations should be considered. Our assessment of hallux valgus and varus knees was based on clinical observations while foot pain and several of the functional outcomes were self-reported, which may have caused misclassification. Besides, for the analysis of varus knee, the control group included both normal and valgus knee. However, our finding also suggested that using simple clinical observations (such as intercondylar distance > 3 cm) can identify people who are at risk of OA. The use of ICD codes to define incident cases of OA may have also led to both false-positives and false-negatives should there be misdiagnosis or recording errors, and the number of incident OA cases in this study was relatively small (N = 33). Furthermore, it should be noted there are no clinically defined cut-off points for MAI and CEPI. Hence, while we followed previous studies to define foot posture and function based on the top and bottom quintiles of the MAI and CEPI values [20], our results may not be directly generalizable to other populations. Finally, as in other observational studies, our results do not necessarily infer causality. Further longitudinal and clinical studies are therefore needed to confirm our findings and examine if interventions on varus alignment, such as weight reduction or high tibial osteotomy [38], may prevent the development of OA.

## 5 Conclusions

In conclusion, foot pain, but not foot deformities, is associated with functional limitations and recurrent falls in community-dwelling Chinese adults. Moreover, varus knee is associated with an increased risk of incident OA, highlighting knee malalignment as a risk factor for OA in the population.

## CRediT author statement

**Jonathan K. L. Mak**: Conceptualization, Formal analysis, Methodology, Writing - original draft. **Kathryn Choon Beng Tan**: Data curation, Writing - review & editing. **Janus Siu Him Wong**: Writing - review & editing. **Martin Man Ho Chung**: Writing - review & editing. **Ching-Lung Cheung**: Conceptualization, Data curation, Methodology, Writing - review & editing.

## Conflicts of interest

The authors declare no competing interests.
